# Congenital Idiopathic Cerebro-Spinal Meningitis

**Published:** 1857-12

**Authors:** M. Stoltz


### CONGENITAL IDIOPATHIC CEREBRO-SPINAL MENINGITIS, IN THE HOSPITAL DE LA MATERNITE, STRASBOURG.

BY M. STOLTZ.

The following case is very interesting in a diagnostic point of view, and especially its anatomical lesions, as they completely resemble the appearances observed in the victims of the epidemic of meningitis, which prevailed some years ago in Strasbourg. M. P.—a woman, 37 years of age, servant, entered the hospital, Dec. 28, 1856. She was of moderate stature, had black hair, sanguine temperament and good constitution. She was delivered at the age of 25 years, of a boy, which she nursed two years, and is now living. Her present pregnancy has not been attended with any of the usual sympathetic symptoms. At the commencement she had tertian intermittent fever, which was easily cured by sulphate of quiniæ. The 21st of January, 1857, without apparent cause, she lost a large quantity of liquor amni with considerable meconium. Almost at the same time she ceased to feel the movements of the fœtus, although the heart could be heard beating with increased rapidity. In view of these circumstances, the accomplished attendant, M'lle Laugel, diagnosticated a congenital disease of the child. The child presented in the first position, and was promptly expelled on the 23d of January, after ten hours' labor. It was apparently dead from asphyxia. Respiration, after a long time, was established with great difficulty, and in an imperfect manner. At length, the child uttered a faint and mournful cry, the skin grew warm, and color insensibly pervaded the surface. On the 25th the child did not suck, nor swallow anything given from a spoon, as everything put in its mouth ran out again; was drowsy constantly, and the face of a yellow color. It does not cry, and utters no sound but a faint, constantly-repeated moan. The meconium was discharged once during the day. Respiration is short and frequent, but no abnormal sounds are revealed by auscultation. The 26th January, the child has not yet sucked, and takes anything from the spoon with great difficulty and in very small quantities; still moans almost imperceptibly; general rigidity of the limbs; the surface natural in color; the mouth can be easily opened; no trismus.

*January 27th.*—The child did nothing but moan throughout the night; it would not take the breast, nor nourishment from the spoon; has passed more meconium. There are no muscular contractions nor paralysis observable. The cranium is remarkably small, with considerable depression above the temples. The eyes are gently closed, but may be opened; the head forcibly thrown backward, and when the head is seized, it feels as if a part of the column (opisthotonos). Two leeches to be applied, one on each side of the vertebral column between the shoulders; ten parts of olive oil to two parts chloroform, mixed thoroughly for liniment, to be rubbed along the whole length of the spine. *28th.*—The condition of the child is unchanged; it has moaned constantly during the day; two alvine evacuations of a green color; the leeches were borne well. This morning there is less rigidity of the spinal column, but lockjaw and contraction of the muscles of the upper and lower extremities, with occasional nervous trembling, resembling successive shocks of electricity. The eyes are less rigidly closed, but look hollow and haggard. Respiration is almost completely abdominal, and deglution almost impossible, a few drops of fluid only finding its way into the stomach at each effort. Frictions of chloroform to spine, cataplasm to the legs; one grain of musk, suspended in one ounce of mucilage, to be given in teaspoonful dose immediately. It could not take the medicine, and remained in pretty much the same condition until seven o'clock in the evening, when it died.

*Autopsy, January 30th.*—Cadaveric rigidity very great; fingers strongly flexed on the palms of the hands covering the thumb. The palmar surface of the hands presented deep indentations, produced by the pressure of the ends of the fingers. The feet were strongly flexed upon the leg, the two forming an acute angle with each other; the great toes were also equally firmly bent upon the foot. Nowhere upon the surface was perceptible either hardness or infiltration of the cellular tissue. Upon opening the skull, besides a quantity of serous effusion of a yellow color between the pia mater and arachnoid, there was a gelatinous exudation quite abundant along each side of the superior longitudinal sinus. The vein which emptied into this sinus, as well as all the others in the neighborhood, were gorged with blood. The same sort of exudation existed also in great abundance between the two hemispheres and their outer borders, but in less quantity. The ventricles were dilated with limpid serum. The choroid plexus was covered with pseudomembranous deposits perfectly organized, but in which the microscope revealed no pus globules. The same gelatinous exudation observed on the upper surface was spread out on the middle and posterior lobes. It was also found in great abundance on the cerebellum and vermiform process. It was also prolonged upon the pons varoli and medulla oblongata. Abundant pus globules were detected in the substance of the vermifor process of the cerebellum. The veins on the base of the brain, and those passing out of cranium, were greatly enlarged and engorged with black blood. The cerebral substance itself was much more dense than in young infants generally.

On opening the spinal membranes, an abnormal vascularity of the pia mater was shown—the longitudinal rachidian sinuses were so large as to resemble in volume the veins on the base of the cranium. In the cervical portion of the medulla spinalis there was no inflammatory exudation; it did not make its appearance above the lower dorsal region, whence it continued very abundant down to the canda equina. The viscera of the thoracic and abdominal cavities were normal in their appearance. The lungs might perhaps have been a little too red around the lower edge, also the liver and spleen. There was nothing notable in any of the other organs. The disease was congenital. It commenced in the mother's womb. * * * * *

Instances may be found in the works of different authors of infants when born being affected with sleeroderma, erysipelas, peritonitis, pleuritis, as also cases of meningitis, consequent to an operation for spina-bifida; but we believe there is no case on record so well attested by the symptoms and microscopic lesions of congenital, idiopathic cerebro-spinal meningeal inflammation, as this one.

This is the only case that has presented itself in the extensive practice of Professor Stoltz.—*Gazette des Hopitaux.*

